# Supplementary material for: Adoptive immunotherapy with transient anti-CD4 treatment enhances anti-tumor response by increasing IL-18Rαhi CD8+ T cells
Source: Nat Commun. 2021 Sep 7;12:5314. doi: 10.1038/s41467-021-25559-7 (PMC8423719; doi:10.1038/s41467-021-25559-7)
Supplement: Supplementary file 1 — Supplementary Information [file 41467_2021_25559_MOESM1_ESM.pdf]

Adoptive immunotherapy with transient anti-CD4 treatment enhances anti-tumor response by increasing IL-18Rα^{hi} CD8^{+} T cells

Kim et al.

Supplementary Information

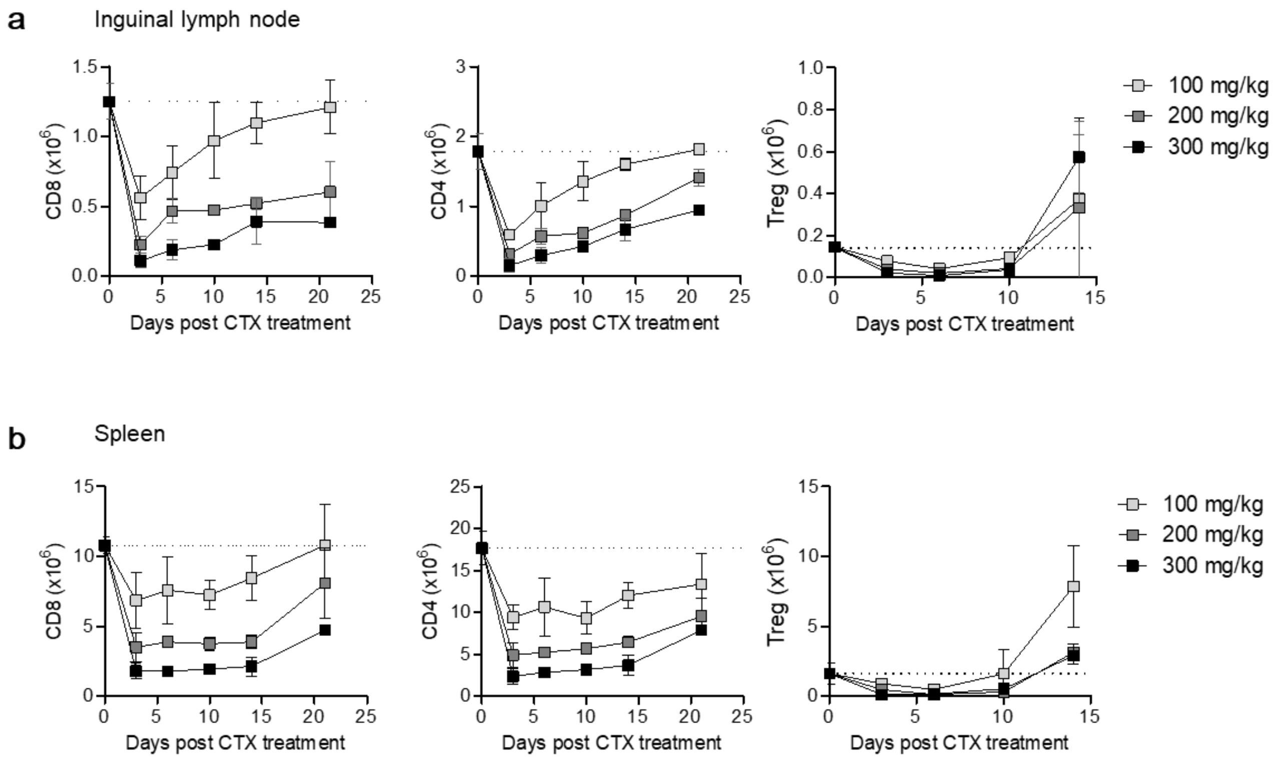

Supplementary Figure 1. The number of CD4^{+} or CD8^{+} cells after cyclophosphamide (CTX) treatment. Numbers of CD8^{+} T (left), CD4^{+} T (middle), and regulatory T (Treg) cells (right) from inguinal lymph node (**a**) and spleen (**b**) of CTX-treated C57BL/6 mice are shown. Mice were intraperitoneally injected with the indicated doses (mg/kg) of CTX as shown in Fig. 1a. n=3 mice per group. Error bars indicate mean ± SD. Source data are provided as a Source Data file.

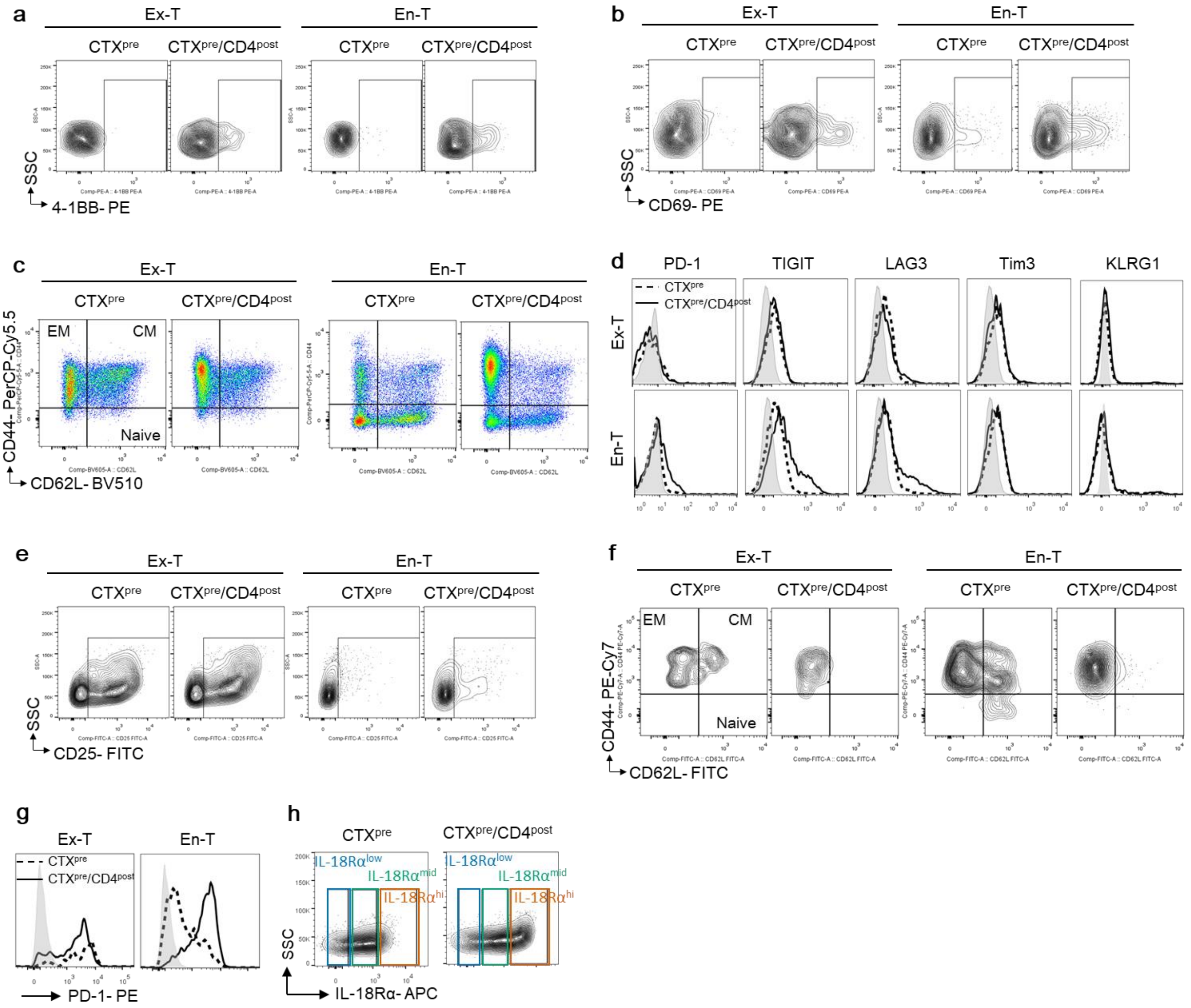

Supplementary Figure 2. Phenotypical characteristics of cells that experienced the CTX^{pre}/CD4^{post} regimen. (**a-d**) Representative flow cytometry images of *ex vivo* primed Thy1.1^{+} Pmel-1 CD8^{+} T (ex-T) and endogenous CD8^{+} T (en-T) cells in lymphoid tissues. 4-1BB (**a**) and CD69 (**b**) expression are shown (Fig. 2b). (**c**) Naïve, EM (effector memory), and CM (central memory) subsets are indicated in the flow cytometry images (Fig. 2c). (**d**) Expression levels of representative inhibitory receptors are shown (Fig. 2d). (**e**) Representative flow cytometry images of CD25 expression (Fig. 3c). (**f, g**) Representative flow cytometry images showing CD44/CD62L (**f**) and PD-1 expression (**g**) in ex-T and en-T cells in tumor tissues (Fig. 3h, i). (**h**) Representative IL-18Rα expression in ex-T cells in lymphoid tissues (Fig. 5d). CTX^{pre}, cyclophosphamide pre-conditioning; CD4^{post}, anti-CD4 post-conditioning.

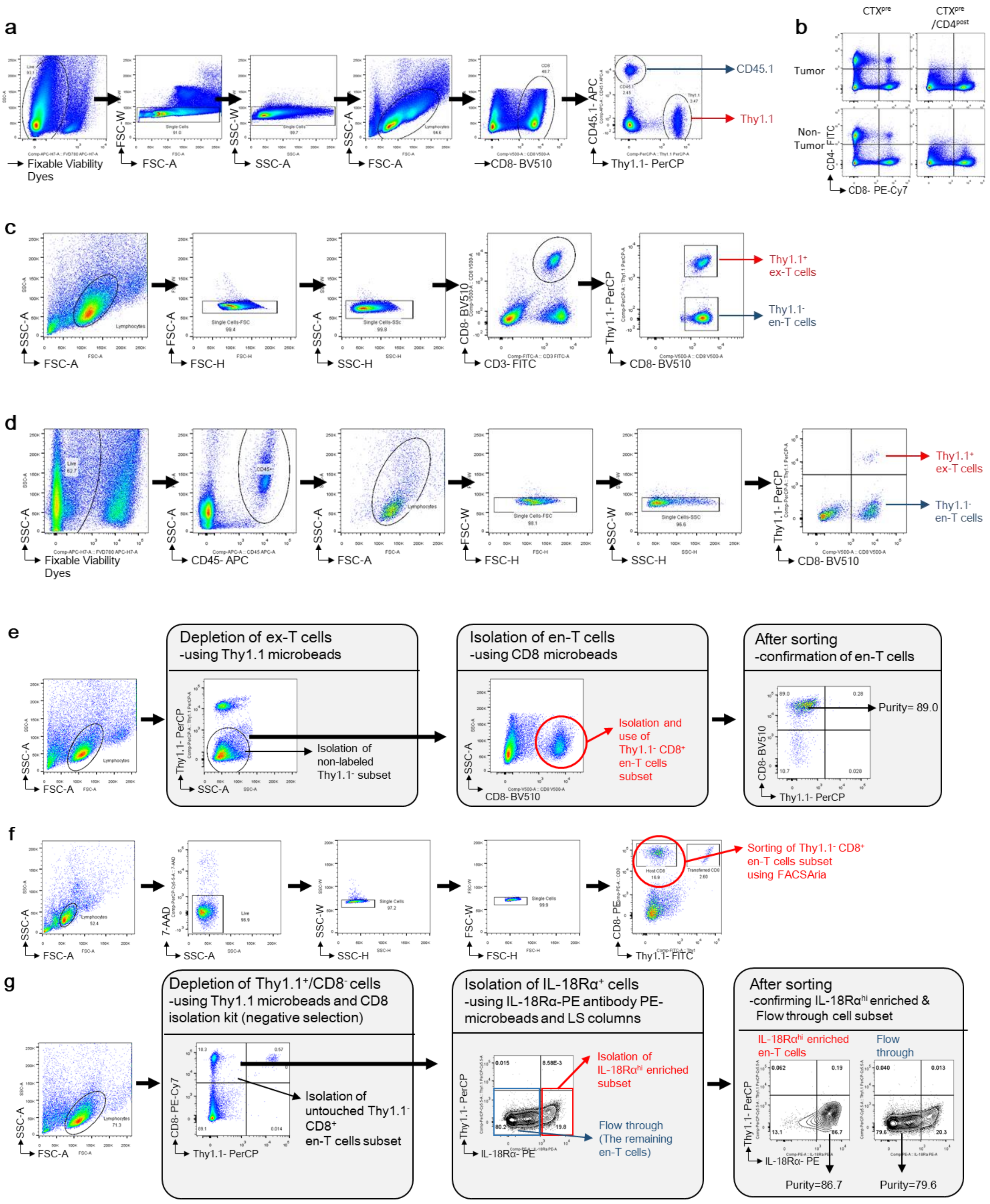
a
Fixable Viability Dyes
FSC-W
FSC-A
SSC-W
SSC-A
SSC-A
FSC-A
CD8- BV510
CD45.1- APC
Thy1.1- PerCP
CD45.1
Thy1.1
b
CTXpre
CTXpre /CD4post
Tumor
Non-Tumor
CD4- FITC
CD8- PE-Cy7
c
SSC-A
FSC-A
FSC-A
FSC-H
SSC-A
SSC-H
CD8- BV510
CD3- FITC
Thy1.1- PerCP
CD8- BV510
Thy1.1+ ex-T cells
Thy1.1- en-T cells
d
SSC-A
Fixable Viability Dyes
SSC-A
CD45- APC
SSC-A
FSC-A
FSC-W
FSC-H
SSC-W
SSC-H
Thy1.1- PerCP
CD8- BV510
Thy1.1+ ex-T cells
Thy1.1- en-T cells
e
SSC-A
FSC-A
Depletion of ex-T cells
-using Thy1.1 microbeads
Thy1.1- PerCP
SSC-A
Isolation of non-labeled Thy1.1- subset
Isolation of en-T cells
-using CD8 microbeads
SSC-A
CD8- BV510
Isolation and use of Thy1.1- CD8+ en-T cells subset
After sorting
-confirmation of en-T cells
CD8- BV510
Thy1.1- PerCP
Purity= 89.0
f
SSC-A
FSC-A
7-AAD
SSC-A
SSC-W
SSC-H
FSC-W
FSC-H
CD8- PE
Thy1.1- FITC
Sorting of Thy1.1- CD8+ en-T cells subset using FACSAria
g
SSC-A
FSC-A
Depletion of Thy1.1+/CD8- cells
-using Thy1.1 microbeads and CD8 isolation kit (negative selection)
CD8- PE-Cy7
Thy1.1- PerCP
Isolation of untouched Thy1.1- CD8+ en-T cells subset
Isolation of IL-18Rα+ cells
-using IL-18Rα-PE antibody PE-microbeads and LS columns
Thy1.1- PerCP
IL-18Rα- PE
Isolation of IL-18Rαhi enriched subset
Flow through (The remaining en-T cells)
After sorting
-confirming IL-18Rαhi enriched & Flow through cell subset
IL-18Rαhi enriched en-T cells
Flow through
Thy1.1- PerCP
IL-18Rα- PE
Purity=86.7
Purity=79.6

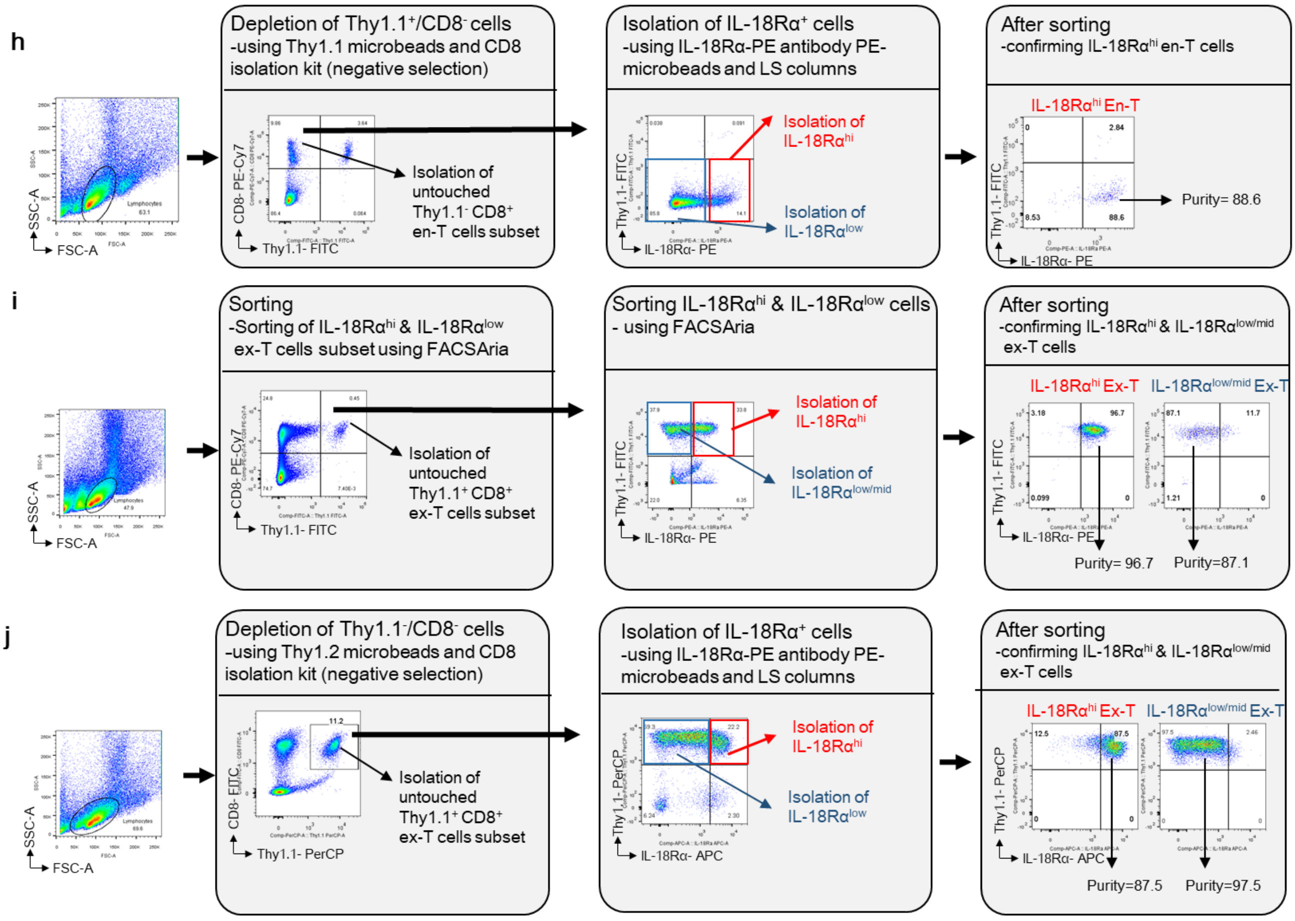

Supplementary Figure 3. Gating and sorting strategies for evaluating *ex vivo* primed and endogenous CD8$^+$ T cells. (**a**) The indicated gating strategy was applied to define *ex vivo* primed Thy1.1$^+$ Pmel-1 CD8$^+$ T (ex-T) and endogenous CD8$^+$ T (en-T) cells in Fig. 2e-j and Supplementary Fig. 4. Viable CD8$^+$ T cells were defined as viability dye-negative and CD8-positive subset. Cells were partitioned into Thy1.1$^+$ Pmel-1 CD8$^+$ T cells (served as ex-T) and CD45.1$^+$ CD8$^+$ T cell (served as en-T) subsets using Thy1.1- and CD45.1-specific antibodies. (**b**) Representative staining of CD4$^+$ and CD8$^+$ T cells in the inguinal lymph nodes of the mice shown in Fig. 2e-j. (**c**) The indicated gating strategy was applied to define ex-T and en-T cells in lymphoid tissues (Figs. 2-6). (**d**) The indicated gating strategy was applied to define ex-T and en-T cells in tumor tissues (Fig. 3f-i). (**e-j**) Cell sorting strategy to separate the indicated en-T or ex-T cell subsets. (**e**) Microbead-based sorting was performed to isolate en-T cells in Fig. 3d. (**f**) FACS-based sorting was performed to isolate en-T cells in Figs. 3e and 4b. (**g**) Microbead-based sorting was performed to enrich IL-18Rα^{hi} en-T cells in Fig. 4d-f. (**h**) Microbead-based sorting was performed to enrich IL-18Rα^{hi} en-T cells in Fig. 5b, c. (**i**) FACS-based sorting was performed to isolate IL-18Rα^{hi} and IL-18Rα$^{low/mid}$ ex-T cells in Fig. 5f. (**j**) Microbead-based sorting was performed to separate IL-18Rα^{hi} ex-T cells and IL-18Rα$^{low/mid}$ ex-T cells in Fig. 5g, h. The purity was determined by calculating the ratio of the desired cell fraction among the parent population.

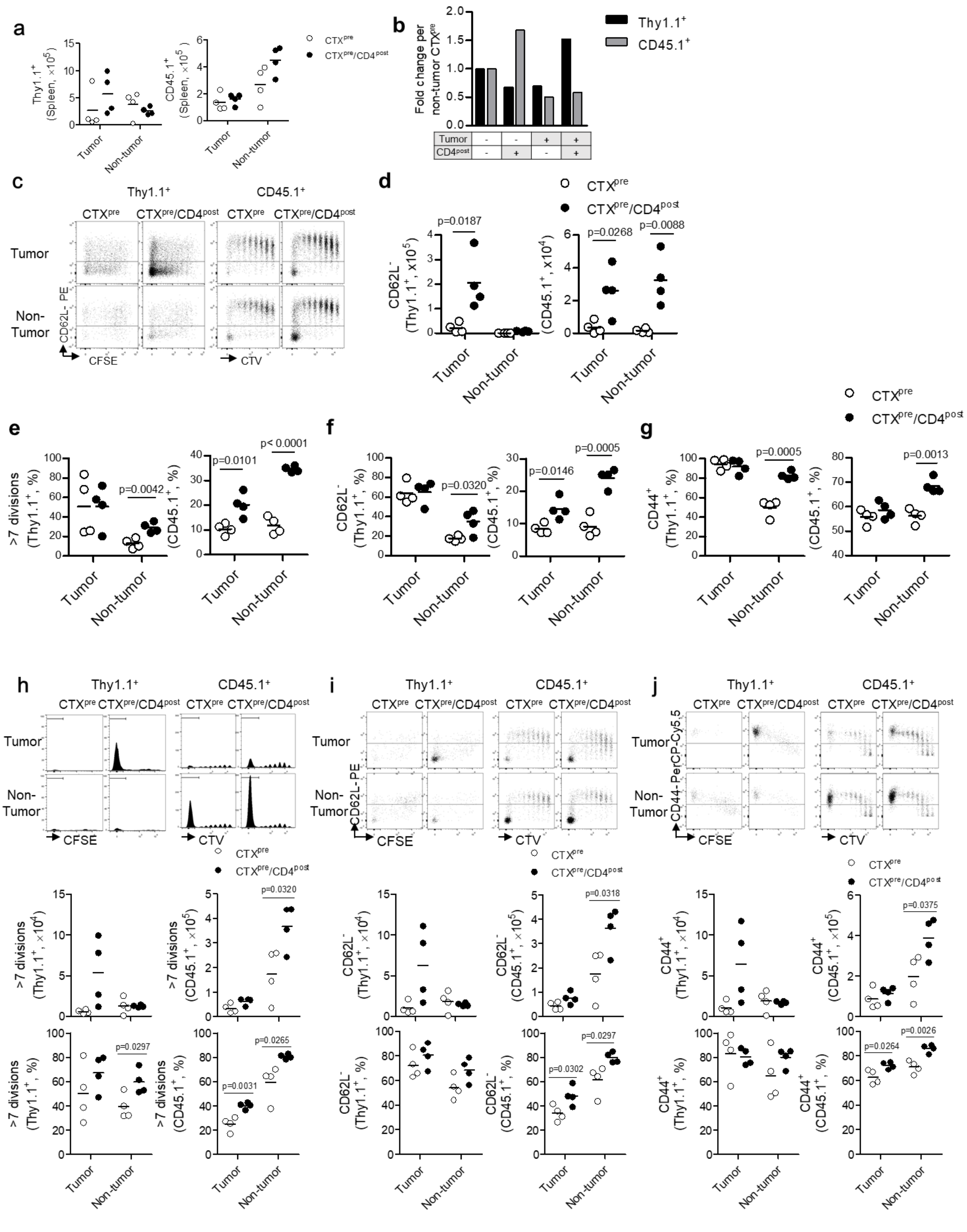

Supplementary Figure 4. Analysis of $CD8^{+}$ T cell proliferation and differentiation in lymphoid tissues following anti-CD4 post-conditioning. Evaluation of the proliferation of *ex vivo* primed $Thy1.1^{+}$ Pmel-1 $CD8^{+}$ T ($Thy1.1^{+}$) cells and

polyclonal $CD8^+$ T ($CD45.1^+$) cells in the presence or absence of tumor antigen as shown in Fig. 2e. (**a**) The numbers of $Thy1.1^+$ and $CD45.1^+$ cells in spleen were calculated as in lymph nodes (Fig. 2f). (**b**) The fold-change in cell number in the spleen compared with the average of the non-tumor CTX^{pre} group is shown, similar to that in lymph nodes (Fig. 2g). (**c-g**) Supplementary data of lymph node analysis shown in Fig. 2h-j. (**c**) Representative flow cytometry images of $Thy1.1^+$ and $CD45.1^+$ cells analyzed for CD62L expression. (**d**) The number of $Thy1.1^+$ and $CD45.1^+$ cells without CD62L expression was calculated. (**e-g**) The frequency of $Thy1.1^+$ and $CD45.1^+$ cells with indicated cell divisions (**e**) and marker expression (**f, g**) were analyzed. (**h-j**) Analysis of proliferation/differentiation of $CD8^+$ T cells in the spleen (as analyzed in the inguinal lymph nodes in Fig. 2h-j and Supplementary Fig. 4c-g). n=4 mice per group. Each symbol indicates the calculated value of an individual mouse. Horizontal bars indicate means. Two-tailed unpaired Student's t-test. CTX^{pre}, cyclophosphamide pre-conditioning; $CD4^{post}$, anti-CD4 post-conditioning; CFSE, carboxyfluorescein succinimidyl ester; CTV, CellTrace Violet. Source data are provided as a Source Data file.

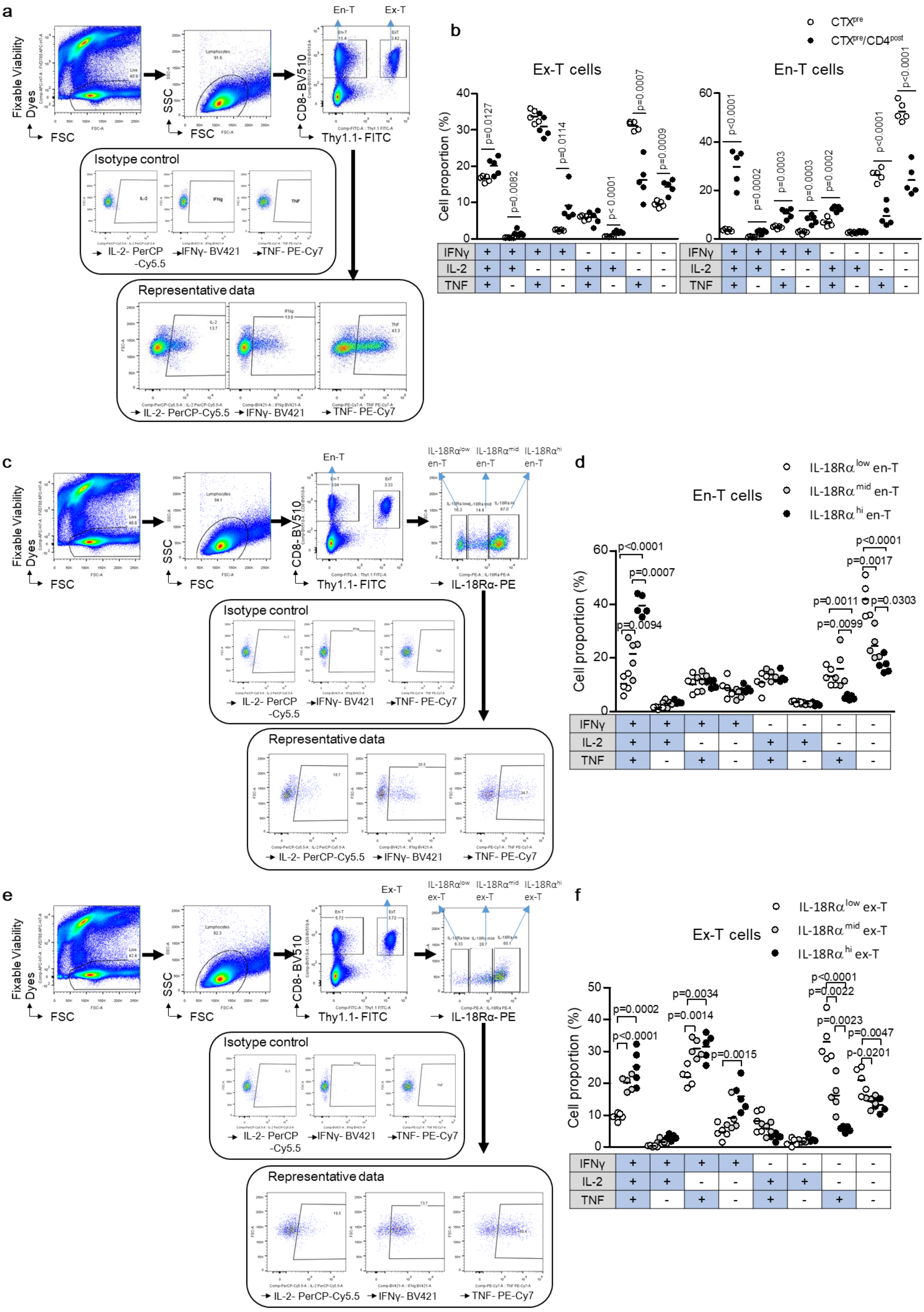
a
Fixable Viability Dyes
FSC
SSC
CD8- BV510
Thy1.1- FITC
En-T
Ex-T
Isotype control
IL-2- PerCP -Cy5.5
IFNγ- BV421
TNF- PE-Cy7
Representative data
IL-2- PerCP-Cy5.5
IFNγ- BV421
TNF- PE-Cy7
b
CTXpre
CTXpre/CD4post
Ex-T cells
En-T cells
Cell proportion (%)
p=0.0127
p=0.0082
p=0.0114
p< 0.0001
p=0.0007
p=0.0009
p<0.0001
p=0.0002
p=0.0003
p=0.0003
p=0.0002
p<0.0001
p<0.0001
IFNγ + + + + - - - -
IL-2 + + - - + + - -
TNF + - + - + - + -
c
Fixable Viability Dyes
FSC
SSC
CD8- BV510
Thy1.1- FITC
En-T
IL-18Rαlow en-T
IL-18Rαmid en-T
IL-18Rαhi en-T
IL-18Rα- PE
Isotype control
IL-2- PerCP -Cy5.5
IFNγ- BV421
TNF- PE-Cy7
Representative data
IL-2- PerCP-Cy5.5
IFNγ- BV421
TNF- PE-Cy7
d
En-T cells
IL-18Rα low en-T
IL-18Rα mid en-T
IL-18Rα hi en-T
Cell proportion (%)
p<0.0001
p=0.0007
p=0.0094
p<0.0001
p=0.0017
p=0.0011
p=0.0303
p=0.0099
IFNγ + + + + - - - -
IL-2 + + - - + + - -
TNF + - + - + - + -
e
Fixable Viability Dyes
FSC
SSC
CD8- BV510
Thy1.1- FITC
Ex-T
IL-18Rαlow ex-T
IL-18Rαmid ex-T
IL-18Rαhi ex-T
IL-18Rα- PE
Isotype control
IL-2- PerCP -Cy5.5
IFNγ- BV421
TNF- PE-Cy7
Representative data
IL-2- PerCP-Cy5.5
IFNγ- BV421
TNF- PE-Cy7
f
Ex-T cells
IL-18Rα low ex-T
IL-18Rα mid ex-T
IL-18Rα hi ex-T
Cell proportion (%)
p=0.0002
p<0.0001
p=0.0034
p=0.0014
p=0.0015
p<0.0001
p=0.0022
p=0.0023
p=0.0047
p=0.0201
IFNγ + + + + - - - -
IL-2 + + - - + + - -
TNF + - + - + - + -

Supplementary Figure 5. Analysis of the polyfunctionality of CD8$^+$ T cells. *Ex vivo* primed tumor-reactive CD8$^+$ T (ex-T) and endogenous CD8$^+$ T (en-T) cells isolated from CTX^{pre}- or CTX^{pre}/CD4^{post}-experienced mice were stimulated with the Cell Stimulation Cocktail for 6 h; IL-2, IFN-γ, and TNF expression were then determined using intracellular cytokine staining. The polyfunctionality of ex-T/en-T cells (a, b), IL-18Rα^{hi}/IL-18Rα^{mid}/IL-18Rα^{low} en-T cells (c, d), and IL-18Rα^{hi}/IL-18Rα^{mid}/IL-18Rα^{low} ex-T cells (e, f) were evaluated. (**a, c, e**) Gating strategy for evaluating cytokine expression in ex-T/en-T cells. The IL-2, IFN-γ, and TNF expression were determined using isotype control. IL-18Rα^{hi}/IL-18Rα^{mid}/IL-18Rα^{low} en-T cells from CTX^{pre}/CD4^{post}-experienced mice (**c**), and IL-18Rα^{hi}/IL-18Rα^{mid}/IL-18Rα^{low} ex-T cells from CTX^{pre}/CD4^{post}-experienced mice (**e**) were used. (**b, d, f**) Results of flow cytometry analyses. Boolean gating was applied to determine the proportion of cells with eight cytokine expression combinations. n=5 biologically independent animals. Error bars indicate means ± SD. Two-tailed unpaired Student's t-test. CTX^{pre}, cyclophosphamide pre-conditioning; CD4^{post}, anti-CD4 post-conditioning. Source data are provided as a Source Data file.

a

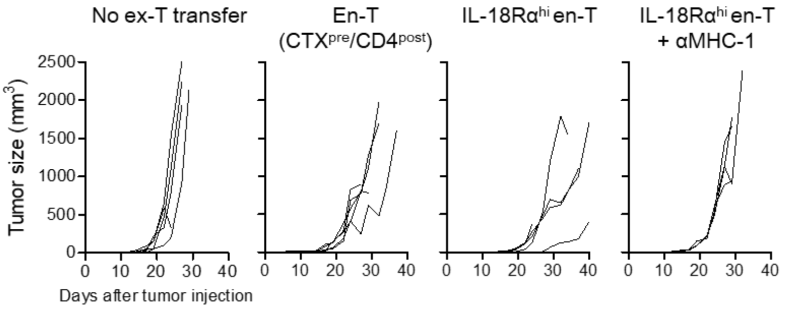

b

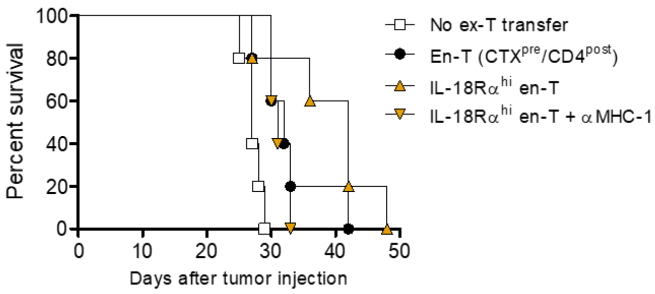

c

	No ex-T transfer	En-T (CTX^{pre} /CD4^{post})	IL-18Rα^{hi} en-T	IL-18Rα^{hi} en-T + αMHC-1
No ex-T transfer		N.D	N.D	N.D
En-T (CTX^{pre}/CD4^{post})			**	***
IL-18Rα^{hi} en-T				***
IL-18Rα^{hi} en-T + αMHC-1				

Supplementary Figure 6. Anti-tumor effect of IL-18Rα^{hi} endogenous CD8$^+$ T cells. Endogenous CD8$^+$ T (en-T) cells (1 × 10^6) from CTX^{pre}/CD4^{post}-experienced mice were transferred to CTX^{pre}-experienced melanoma-bearing *Rag1* knock-out mice (Fig. 5b, c). IL-18Rα^{hi} en-T means magnetically enriched cells as in Supplementary Fig. 3h. (**a**) Tumor growth curves in Fig. 5c were converted into longitudinal changes of an individual mouse. (**b**) Survival rate per group is shown. (**c**) Statistical analysis of short-term (~27 days) tumor suppression was performed using two-way ANOVA with the Bonferroni post hoc test. *, $p<0.05$; **, $p<0.01$; ***, $p<0.001$. n=5 mice per group. CTX^{pre}, cyclophosphamide pre-conditioning; CD4^{post}, anti-CD4 post-conditioning; αMHC-1, anti-MHC-1 blocking antibody; N.D, not determined. Source data are provided as a Source Data file.

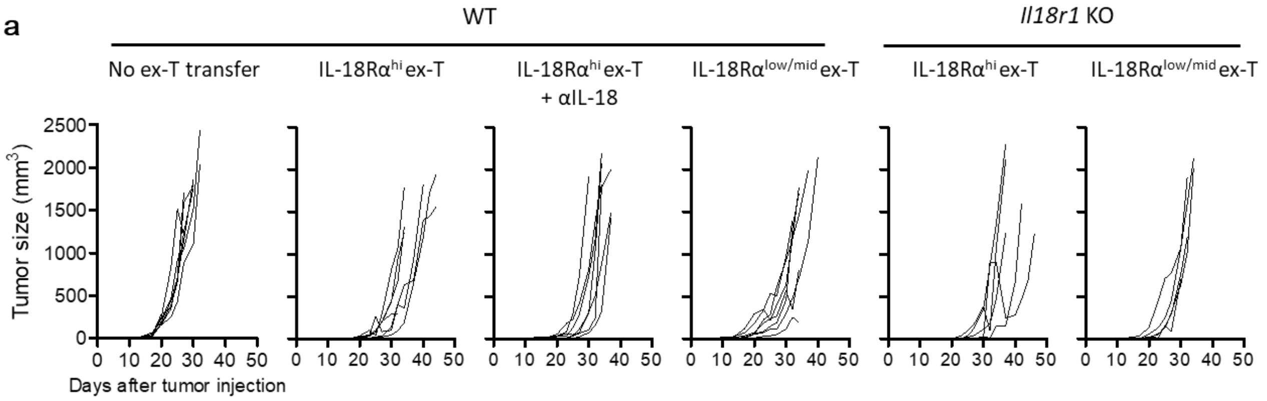

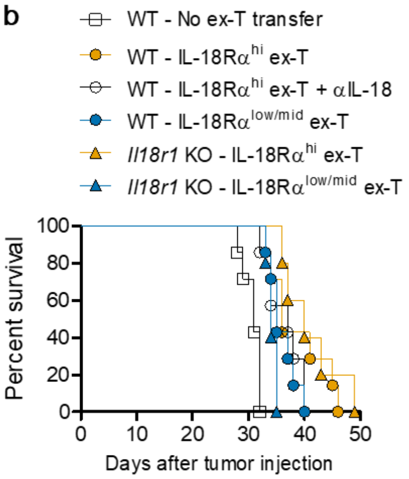

c

	WT No ex-T transfer	WT IL-18Rα^{hi} ex-T	WT IL-18Rα^{hi} ex-T + αIL-18	WT IL-18Rα^{low} ex-T	*Il18r1* KO IL-18Rα^{hi} ex-T	*Il18r1* KO IL-18Rα^{low} ex-T
WT - No ex-T transfer		N.D	N.D	N.D	N.D	N.D
WT- IL-18Rα^{hi} ex-T			*	*	ns	***
WT- IL-18Rα^{hi} ex-T + αIL-18				ns	**	ns
WT- IL-18Rα^{low} ex-T					***	***
Il18r1 KO IL-18Rα^{hi} ex-T						***
Il18r1 KO IL-18Rα^{low} ex-T						

Supplementary Figure 7. Evaluation of anti-tumor effect of IL-18Rα^{hi} *ex vivo* primed tumor-reactive CD8^{+} T cells. *Ex vivo* primed tumor-reactive CD8^{+} T (ex-T) cells (5 × 10^{5}) from CTX^{pre}/CD4^{post}-experienced mice were transferred to CTX^{pre}-experienced melanoma-bearing wild-type (WT) or *Il18r1* knock-out mice (Fig. 5g, h) IL-18Rα^{hi} and IL-18Rα$^{low/mid}$ ex-T cells were magnetically sorted as in Supplementary Fig. 3j. (**a**) Tumor growth curves in Fig. 5h were converted into longitudinal changes of an individual mouse. (**b**) Survival rate per group is shown. (**c**) Statistical analysis of short-term (~32 days) tumor suppression was performed using two-way ANOVA with the Bonferroni post hoc test. *, $p<0.05$; **, $p<0.01$; ***, $p<0.001$. WT groups, n=7 mice/group; *Il18r1* knock-out groups, n=5 mice/group. CTX^{pre}, cyclophosphamide pre-conditioning; CD4^{post}, anti-CD4 post-conditioning; αIL-18, IL-18 neutralizing antibody; N.D, not determined. Source data are provided as a Source Data file.

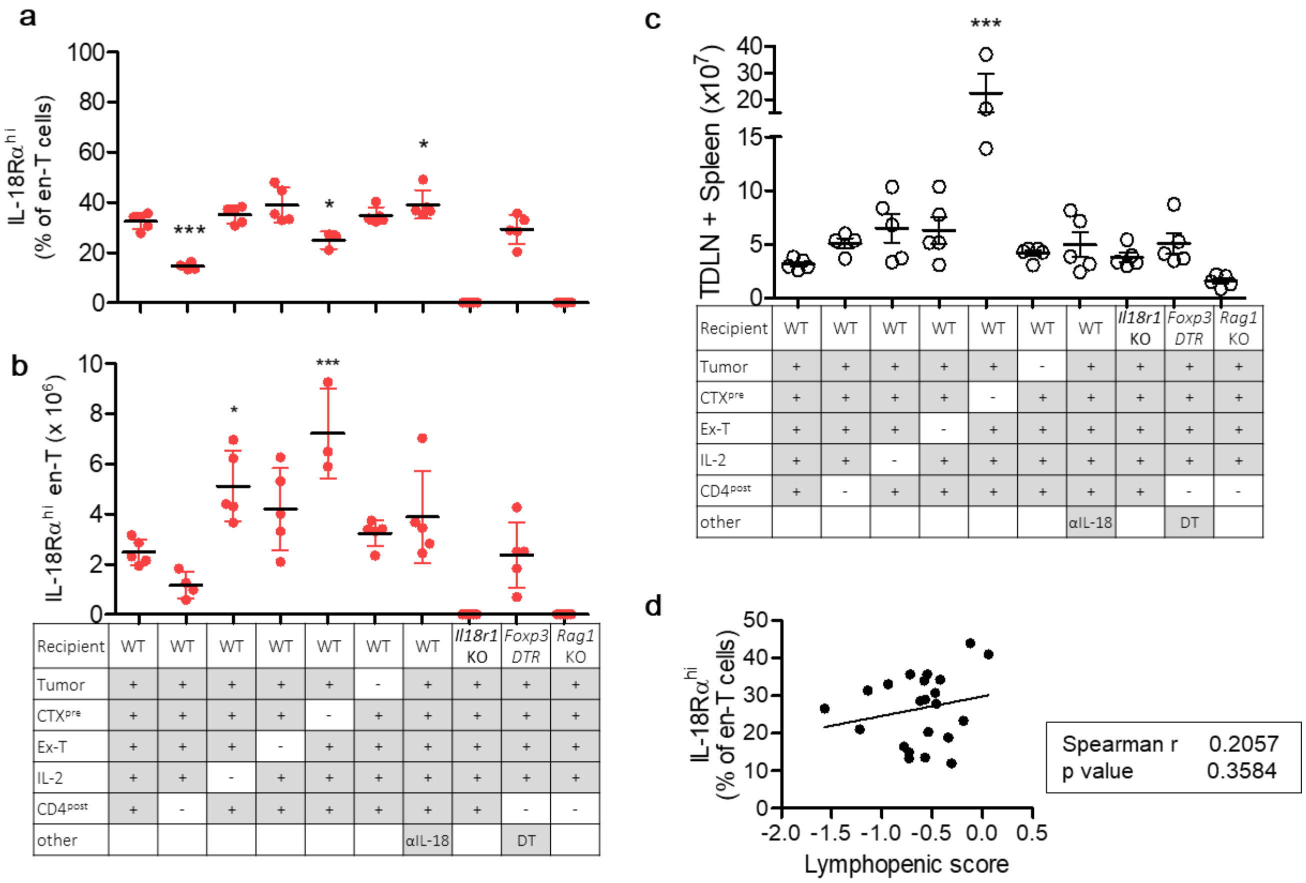

Supplementary Figure 8. Effect of various factors on expansion of IL-18Rα^{hi} CD8^{+} T cells. The treatment with full regimen (as in Fig. 4a) or a modified regimen that lacks each variable was applied to mice (n=5 mice per group) as in Fig. 6a, b. On days 25, the ratio (**a**) and the number (**b**) of IL-18Rα^{hi} endogenous CD8^{+} T (en-T) cells in lymphoid tissues were analyzed. (**c**) The absolute count of cells in inguinal lymphoid nodes and spleen is shown. (**d**) The correlation between lymphopenic score (represented as −log [total cell count in spleen and tumor-draining lymph nodes]) and the proportion of IL-18Rα^{hi} en-T cells was shown. Each symbol indicates the calculated value of an individual mouse. Error bars indicate means ± SD. *, $p<0.05$; ***, $p<0.001$; one-way ANOVA with the Dunnett's post hoc test (**a-c**; the 1st group was the control) and two-tailed Spearman's rank correlation (**d**) were used to determine statistical significance. Ex-T, *ex-vivo* primed melanoma-reactive CD8^{+} T cells; CTX^{pre}, cyclophosphamide pre-conditioning; CD4^{post}, anti-CD4 post-conditioning; αIL-18, IL-18 neutralizing antibody; DT, diphtheria toxin; WT, wild-type mice; TDLN, tumor-draining lymph node. Source data are provided as a Source Data file.

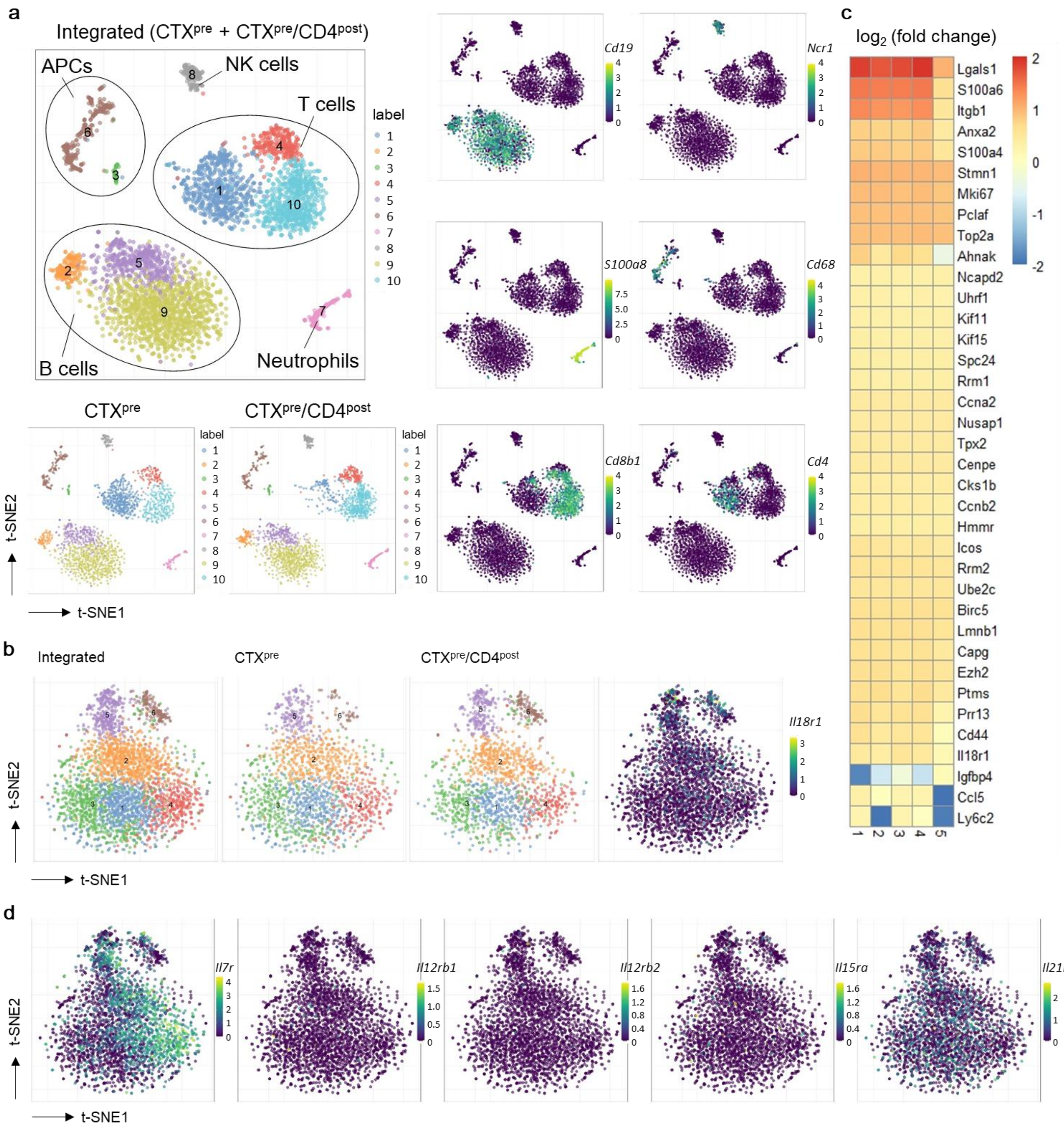

Supplementary Figure 9. Single-cell transcriptome analysis of lymphoid tissues and endogenous CD8$^+$ T cells. Visualization of single-cell transcriptome analysis of CTX^{pre} and CTX^{pre}/CD4^{post}-experienced lymphoid tissues and endogenous CD8$^+$ T (en-T) cells. (**a**) The analysis of cells from whole lymphoid tissues. After unsupervised clustering, cells were projected on two-dimensional space using t-distributed stochastic neighbor embedding (t-SNE). The information related to groups, clusters, and the indicated gene expression are shown. (**b-d**) Analysis of en-T cells. (**b**) En-T cells were projected on two-dimensional space. The information related to groups, clusters, and *Il18r1* expression are shown. (**c**) Genes that are differentially expressed compared with cluster 6 are listed. (**d**) The log-normalized transcript levels of the indicated genes are shown. CTX^{pre}, cyclophosphamide pre-conditioning; CD4^{post}, anti-CD4 post-

conditioning; APCs, antigen-presenting cells; NK cells, natural killer cells. Cells from four mice of each group were pooled and analyzed.

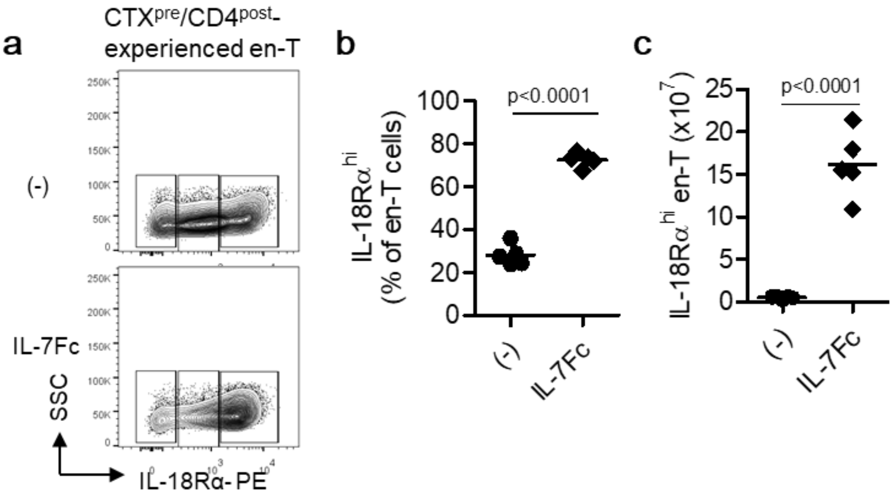

Supplementary Figure 10. Effect of IL-7 signaling on expansion of IL-18Rα^{hi} endogenous CD8^{+} T cells. The experiment was performed as in Fig. 6g. Representative data (**a**), the ratio (**b**) and the number (**c**) of IL-18Rα^{hi} endogenous CD8^{+} T (en-T) cells are shown. n=5 mice per group. Each symbol indicates the calculated value of an individual mouse. Horizontal bars indicate means. Two-tailed unpaired Student's t-test. IL-7-Fc, nonlytic Fc-fused IL-7; CTX^{pre}, cyclophosphamide pre-conditioning; CD4^{post}, anti-CD4 post-conditioning. Source data are provided as a Source Data file.

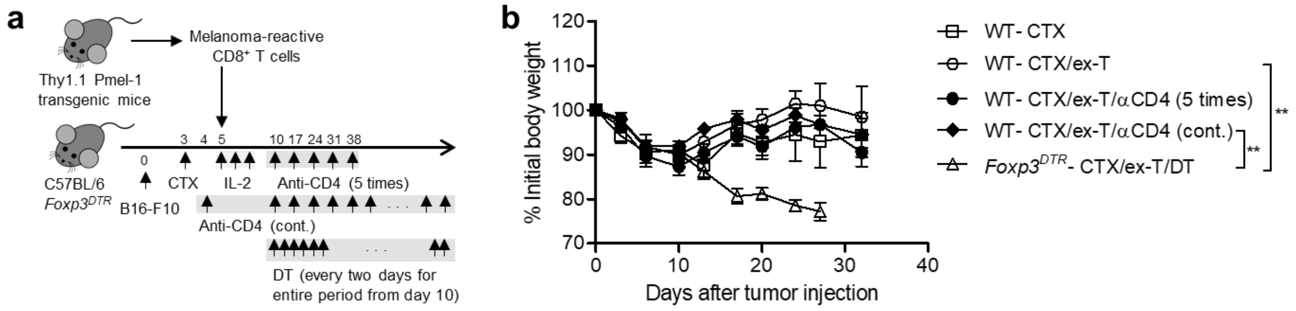

Supplementary Figure 11. Removal of Foxp3+ regulatory T cells leads to weight loss. (**a**) Schematic of the experiment. C57BL/6 or *Foxp3DTR* mice inoculated with 2 × 10^5 B16-F10 melanoma were pre-conditioned with cyclophosphamide (CTX) and infused with 2 × 10^6 *ex vivo* primed Thy1.1+ Pmel-1 CD8+ T (ex-T) cells. Mice with anti-CD4 post-conditioning (αCD4) were treated every week with anti-CD4 antibody from day 10 for 5 weeks (5 times) or continually (cont.). *Foxp3DTR* mice were treated with diphtheria toxin (DT) every two days for the entire period from day 10. (**b**) Longitudinal change of body weight against starting weight of each individual is shown. Error bars indicate means ± SEM; n=5 mice per group. **, p<0.01; two-way ANOVA with the Bonferroni post hoc test. Source data are provided as a Source Data file.

Antibodies for flow cytometry

Specificity	Reactivity	Conjugation	Clone	Manufacturer	Cat #	Dilution
CD3	Mouse	APC-Cy7	145-2C11	BioLegend	100329	1:200
CD4	Mouse	FITC	RM4-4	eBioscience	11-0043-82	1:200
CD4	Mouse	BUV395	RM4-4	BD Biosciences	740208	1:200
CD8a	Mouse	FITC	53-6.7	eBioscience	100706	1:100
CD8a	Mouse	PE	53-6.7	eBioscience	100708	1:200
CD8a	Mouse	APC	53-6.7	eBioscience	17-0071-82	1:200
CD8a	Mouse	PE-Cy7	53-6.7	eBioscience	25-0081-82	1:200
CD8a	Mouse	BV510	53-6.7	BD Biosciences	563068	1:200
CD45	Mouse	APC	30-F11	BD Biosciences	559864	1:200
CD90.1 (Thy1.1)	Mouse	FITC	OX-7	BD Biosciences	554897	1:200
CD90.1 (Thy1.1)	Mouse	APC-Cy7	OX-7	BD Biosciences	561401	1:200
CD90.1 (Thy1.1)	Mouse	PerCP	OX-7	BD Biosciences	557265	1:200
CD90.1 (Thy1.1)	Mouse	BUV496	OX-7	BD Biosciences	741110	1:500
CD45.1	Mouse	APC	A20	BD Biosciences	561872	1:200
CD62L	Mouse	FITC	MEL-14	Tonbo	35-0621	1:100
CD62L	Mouse	BV605	MEL-14	BD Biosciences	563252	1:100
CD44	Mouse	PerCP-Cy5.5	1M7	BD Biosciences	560570	1:100
CD44	Mouse	PE-Cy7	1M7	BD Biosciences	560569	1:100
TIGIT	Mouse	PE	GIGD7	eBioscience	12-9501-80	1:100
CD279 (PD-1)	Mouse	PE	J43	eBioscience	12-9985-82	1:100
CD366 (TIM3)	Mouse	PE	8B.2C12	eBioscience	12-5871-81	1:100
CD223 (LAG-3)	Mouse	PE	C9B7w	eBioscience	12-2231-82	1:100
KLRG1	Mouse	PE	2F1	eBioscience	12-5893-80	1:100
CD25	Mouse	FITC	PC61.5	Invitrogen	MA5-17816	1:100
TNF-alpha	Mouse	PE-Cy7	MP6-XT22	BD Biosciences	561041	1:50
IFNgamma	Mouse	BV421	XMG1.2	BD Biosciences	563376	1:50
IL-2	Mouse	PerCP-Cy5.5	JES6-5H4	BD Biosciences	560544	1:50
CD218a (IL-18Rα)	Mouse	PE	P3TUNYA	eBioscience	12-5183-82	1:100
CD218a (IL-18Rα)	Mouse	APC	P3TUNYA	eBioscience	17-5183-82	1:100
CD314 (NKG2D)	Mouse	PE-CF594	CX5	BD Biosciences	562614	1:100
CD49d	Mouse	BV786	R1-2	BD Biosciences	564397	1:100
T-bet	Mouse	BV650	O4-46	BD Biosciences	564142	1:100
NK1.1	Mouse	BV711	PK136	BioLegend	108745	1:100
CXCR3	Mouse	BV510	CXCR3-173	BioLegend	126528	1:100
4-1BB	Mouse	PE	1AH2	BD Biosciences	558976	1:100
CD69	Mouse	PE	H1.2F3	BD Biosciences	553237	1:100

Antibodies for in vivo treatment

Specificity	Reactivity	Clone	Manufacturer	Cat #	Dilution buffer	Diluted final conc.	Injection volume
Anti-CD4 monoclonal antibody	Mouse	GK1.5	BioXcell	BE0003	InVivoPure™ pH 6.5 Dilution Buffer	2 mg/ml	100 μL(200 μg)
Anti-MHC Class I (H-2) antibody	Mouse	M1/42.3.9.8	BioXcell	BE0077	InVivoPure™ pH 7.0 Dilution Buffer	2 mg/ml	125 μL (250 μg)
Anti-IL-18 monoclonal antibody	Mouse	YIGIF74-1G7	BioXcell	BE0237	InVivoPure™ pH 7.0 Dilution Buffer	2 mg/ml	100 μL(200 μg)

Supplementary Table 1. Antibodies used in this study.
