# Supplementary material for: Adoptive immunotherapy with transient anti-CD4 treatment enhances anti-tumor response by increasing IL-18Rαhi CD8+ T cells
Source: Nat Commun. 2021 Sep 7;12:5314. doi: 10.1038/s41467-021-25559-7 (PMC8423719; doi:10.1038/s41467-021-25559-7)
Supplement: Supplementary file 3 — Description of Additional Supplementary Files [file 41467_2021_25559_MOESM3_ESM.pdf]

Title: Supplementary Data 1.
Description: Comparison of gene-expression profiles between $CTX^{pre}/CD4^{post}$- and CTX^{pre}-experienced endogenous $CD8^{+}$ T cells. Genes with >1.5-fold difference in expression level are listed.

Title: Supplementary Data 2.
Description: Comparison of gene-expression profiles between IL-18Rα^{hi}-enriched and the other endogenous $CD8^{+}$ T cells in the flow through. Genes with >1.5-fold increase in expression level are listed.
