# Supplementary material for: Adoptive immunotherapy with transient anti-CD4 treatment enhances anti-tumor response by increasing IL-18Rαhi CD8+ T cells
Source: Nat Commun. 2021 Sep 7;12:5314. doi: 10.1038/s41467-021-25559-7 (PMC8423719; doi:10.1038/s41467-021-25559-7)
Supplement: Supplementary file 4 — Reporting Summary [file 41467_2021_25559_MOESM4_ESM.pdf]

nature portfolio

Corresponding author(s): Chungyong Han, Beom K. Choi, Byoung S. Kwon

Last updated by author(s): Aug 7, 2021

Reporting Summary

Nature Portfolio wishes to improve the reproducibility of the work that we publish. This form provides structure for consistency and transparency in reporting. For further information on Nature Portfolio policies, see our Editorial Policies and the Editorial Policy Checklist.

Statistics

For all statistical analyses, confirm that the following items are present in the figure legend, table legend, main text, or Methods section.

n/a	Confirmed	
☐	☒	The exact sample size (n) for each experimental group/condition, given as a discrete number and unit of measurement
☐	☒	A statement on whether measurements were taken from distinct samples or whether the same sample was measured repeatedly
☐	☒	The statistical test(s) used AND whether they are one- or two-sided<br>*Only common tests should be described solely by name; describe more complex techniques in the Methods section.*
☒	☐	A description of all covariates tested
☐	☒	A description of any assumptions or corrections, such as tests of normality and adjustment for multiple comparisons
☐	☒	A full description of the statistical parameters including central tendency (e.g. means) or other basic estimates (e.g. regression coefficient) AND variation (e.g. standard deviation) or associated estimates of uncertainty (e.g. confidence intervals)
☐	☒	For null hypothesis testing, the test statistic (e.g. F, t, r) with confidence intervals, effect sizes, degrees of freedom and P value noted<br>*Give P values as exact values whenever suitable.*
☒	☐	For Bayesian analysis, information on the choice of priors and Markov chain Monte Carlo settings
☒	☐	For hierarchical and complex designs, identification of the appropriate level for tests and full reporting of outcomes
☐	☒	Estimates of effect sizes (e.g. Cohen's d, Pearson's r), indicating how they were calculated

Our web collection on statistics for biologists contains articles on many of the points above.

Software and code

Policy information about availability of computer code

Data collection	Microsoft Office Excel 2013, FlowJo v10, GraphPad Prism v5.01, R v4.0.3
Data analysis	The flow cytometric data were analyzed in FlowJo v10 (Tree Star Inc., OR, USA).<br>The statistical data were analyzed in Prism v5.01 GraphPad (CA, USA).<br>Microarray data export processing and analysis was performed using Affymetrix GeneChip Command Console Software and the data were normalized with a robust multi-average method in Affymetrix Power Tools.<br>For TCR repertoire analysis, adapter sequences were removed from raw data using Scythe (v0.994) and Sickle. The processed data were then aligned in MiXCR (https://mixcr.readthedocs.io). Aligned TCRα/β sequences were extracted using VDJtools (https://vdjtools-doc.readthedocs.io).<br>The whole filtering process of bulk RNA-seq analysis was performed using the in-house scripts (TheragenEtex BiO Institute, Suwon, Korea). The filtered reads were mapped to the reference genome (mm10) using the aligner TopHat. Gene expression level was determined using Cufflinks v2.1.1 and visualized using Morpheus (Broad Institute).<br>For single-cell RNA-seq analysis, data processing of the raw data was performed by running the BD Rhapsody Analysis pipelines for sequencing on the Seven Bridges Genomics platform. R version 4.0.3 and batchelor, BiocSingular, edgeR, pheatmap, SingleCellExperiment, scater, scran, and uwot were used to analyze and visualize the data.

For manuscripts utilizing custom algorithms or software that are central to the research but not yet described in published literature, software must be made available to editors and reviewers. We strongly encourage code deposition in a community repository (e.g. GitHub). See the Nature Portfolio guidelines for submitting code & software for further information.

Data

Policy information about availability of data

All manuscripts must include a data availability statement. This statement should provide the following information, where applicable:

- Accession codes, unique identifiers, or web links for publicly available datasets
- A description of any restrictions on data availability
- For clinical datasets or third party data, please ensure that the statement adheres to our policy

The microarray, TCR analysis, bulk and single-cell RNAseq data generated in this study have been deposited in the GEO database under accession code GSE180291, GSE181280, GSE180439, and GSE180991. The remaining data are available within the Article, Supplementary Information or Source Data file.

Field-specific reporting

Please select the one below that is the best fit for your research. If you are not sure, read the appropriate sections before making your selection.

[x] Life sciences [ ] Behavioural & social sciences [ ] Ecological, evolutionary & environmental sciences

For a reference copy of the document with all sections, see nature.com/documents/nr-reporting-summary-flat.pdf

Life sciences study design

All studies must disclose on these points even when the disclosure is negative.

Sample size	Sample sizes were selected based on previous experience with similar type of studies (PMID: 31797935 and 26901151).
Data exclusions	No data was excluded and all outliers were included in data analysis.
Replication	Experimental data reproduced in at least two independent experiments were used to draw conclusions (with the exception of microarray and NGS). The number of repeated experiments is as follows: three times, Figs. 1c-f, 2c, 3b, g-i, 4c, 5d, Supplementary Figs. 5b; twice, Figs. 1a, 2b, d, f-j, 3c, d, 4a, e, g, 5a, c, e, f, h, j, k, 6a-c, h, Supplementary Figs. 1, 4, 5d, f, 6, 7, 8, 10; once, Figs. 3e, 4b, e, f, 6d-f, Supplementary Fig. 9, 11.
Randomization	All experiments were conducted using randomly assigned animals or tissues derived from randomly assigned animals.
Blinding	Most experiments in this study required periodic treatment with agents (e.g. antibody, diphtheria toxin) in the test group. To avoid mistakes occurring during experiments that lasted weeks or months, we conducted non-blinded experiments in the study.

Reporting for specific materials, systems and methods

We require information from authors about some types of materials, experimental systems and methods used in many studies. Here, indicate whether each material, system or method listed is relevant to your study. If you are not sure if a list item applies to your research, read the appropriate section before selecting a response.

Materials & experimental systems

n/a	Involved in the study	
[ ]	[x]	Antibodies
[ ]	[x]	Eukaryotic cell lines
[x]	[ ]	Palaeontology and archaeology
[ ]	[x]	Animals and other organisms
[x]	[ ]	Human research participants
[x]	[ ]	Clinical data
[x]	[ ]	Dual use research of concern

Methods

n/a	Involved in the study	
[x]	[ ]	ChIP-seq
[ ]	[x]	Flow cytometry
[x]	[ ]	MRI-based neuroimaging

Antibodies

Antibodies used

* Antibodies for flow cytometry
CD3 (conjugate, APC-Cy7; clone, 145-2C11; manufacturer, BioLegend; cat#, 100329; dilution, 1:200)
CD4 (conjugate, FITC; clone, RM4-4; manufacturer, eBioscience; cat#, 11-0043-82; dilution, 1:200)
CD4 (conjugate, BUV395; clone, RM4-4; manufacturer, BD Biosciences; cat#, 740208; dilution, 1:200)
CD8a (conjugate, FITC; clone, 53-6.7; manufacturer, BioLegend; cat#, 100706; dilution, 1:200)
CD8a (conjugate, PE; clone, 53-6.7; manufacturer, BioLegend; cat#, 100708; dilution, 1:200)
CD8a (conjugate, APC; clone, 53-6.7; manufacturer, eBioscience; cat#, 17-0071-82; dilution, 1:200)
CD8a (conjugate, PE-Cy7; clone, 53-6.7; manufacturer, eBioscience; cat#, 25-0081-82; dilution, 1:200)

CD8a (conjugate, BV510; clone, 53-6.7; manufacturer, BD Biosciences; cat#, 563068; dilution, 1:200)
CD45 (conjugate, APC; clone, 30-F11; manufacturer, BD Biosciences; cat#, 559864; dilution, 1:200)
Thy1.1 (conjugate, FITC; clone, OX-7; manufacturer, BD Biosciences; cat#, 554897; dilution, 1:200)
Thy1.1 (conjugate, APC-Cy7; clone, OX-7; manufacturer, BD Biosciences; cat#, 561401; dilution, 1:200)
Thy1.1 (conjugate, PerCP; clone, OX-7; manufacturer, BD Biosciences; cat#, 557266; dilution, 1:200)
Thy1.1 (conjugate, BUV496; clone, OX-7; manufacturer, BD Biosciences; cat#, 741110; dilution, 1:500)
CD45.1 (conjugate, APC; clone, A20; manufacturer, BD Biosciences; cat#, 561873; dilution, 1:200)
CD62L (conjugate, FITC; clone, MEL-14; manufacturer, Tonbo; cat#, 35-0621; dilution, 1:100)
CD62L (conjugate, BV605; clone, MEL-14; manufacturer, BD Biosciences; cat#, 563252; dilution, 1:100)
CD44 (conjugate, PerCP-Cy5.5; clone, 1M7; manufacturer, BD Biosciences; cat#, 560570; dilution, 1:100)
CD44 (conjugate, PE-Cy7; clone, 1M7; manufacturer, BD Biosciences; cat#, 560569; dilution, 1:100)
TIGIT (conjugate, PE; clone, GIGD7; manufacturer, eBioscience; cat#, 12-9501-80; dilution, 1:100)
PD-1 (conjugate, PE; clone, J43; manufacturer, eBioscience; cat#, 12-9985-82; dilution, 1:100)
TIM3 (conjugate, PE; clone, 8B.2C12; manufacturer, eBioscience; cat#, 12-5871-81; dilution, 1:100)
LAG-3 (conjugate, PE; clone, C9B7w; manufacturer, eBioscience; cat#, 12-2231-82; dilution, 1:100)
KLRG1 (conjugate, PE; clone, 2F1; manufacturer, eBioscience; cat#, 12-5893-80; dilution, 1:100)
CD25 (conjugate, FITC; clone, PC61.5; manufacturer, Invitrogen; cat#, MA5-17816; dilution, 1:100)
TNF-alpha (conjugate, PE-Cy7; clone, MP6-XT22; manufacturer, BD Biosciences; cat#, 561041; dilution, 1:50)
IFNgamma (conjugate, BV421; clone, XMG1.2; manufacturer, BD Biosciences; cat#, 563376; dilution, 1:50)
IL-2 (conjugate, PerCP-Cy5.5; clone, JES6-5H4; manufacturer, BD Biosciences; cat#, 560544; dilution, 1:50)
IL-18Rα (conjugate, PE; clone, P3TUNYA; manufacturer, eBioscience; cat#, 12-5183-82; dilution, 1:100)
IL-18Rα (conjugate, APC; clone, P3TUNYA; manufacturer, eBioscience; cat#, 17-5183-82; dilution, 1:100)
NKG2D (conjugate, PE-CF594; clone, CX5; manufacturer, BD Biosciences; cat#, 562614; dilution, 1:100)
CD49d (conjugate, BV786; clone, R1-2; manufacturer, BD Biosciences; cat#, 564397; dilution, 1:100)
T-bet (conjugate, BV650; clone, O4-46; manufacturer, BD Biosciences; cat#, 564142; dilution, 1:100)
NK1.1 (conjugate, BV711; clone, PK136; manufacturer, BioLegend; cat#, 108745; dilution, 1:100)
CXCR3 (conjugate, BV510; clone, CXCR3-173; manufacturer, BioLegend; cat#, 126528; dilution, 1:100)
4-1BB (conjugate, PE; clone, 1AH2; manufacturer, BD Biosciences; cat#, 558976; dilution, 1:100)
CD69 (conjugate, PE; clone, H1.2F3; manufacturer, BD Biosciences; cat#, 553237; dilution, 1:100)

* Antibodies for in vivo treatment
anti-mouse CD4 monoclonal antibody (clone, GK1.5; manufacturer, BioXcell; cat#, BE0003)
anti-mouse MHC Class I (H-2) antibody (clone, M1/42.3.9.8; manufacturer, BioXcell; cat#, BE0077)
anti-mouse IL-18 monoclonal antibody (clone, YIGIF74-1G7; manufacturer, BioXCell; cat#, BE0237)

Validation

Antibody profiles, application, and relevant citations are available in The Antibody Registry using the indicated research resource identifiers (RRID).
CD3 (BioLegend; cat#, 100329) RRID: AB_1877171
CD4 (eBioscience; cat#, 11-0043-82) RRID: AB_464900
CD4 (BD Biosciences; cat#, 740208) RRID: AB_2734761
CD8a (BioLegend; cat#, 100706) RRID: AB_312745
CD8a (BioLegend; cat#, 100708) RRID: AB_312747
CD8a (eBioscience; cat#, 17-0071-82) RRID: AB_469335
CD8a (eBioscience; cat#, 25-0081-82) RRID: AB_469584
CD8a (BD Biosciences; cat#, 563068) RRID: AB_2687548
CD45 (BD Biosciences; cat#, 559864) RRID: AB_398672
Thy1.1 (BD Biosciences; cat#, 554897) RRID: AB_395588
Thy1.1 (BD Biosciences; cat#, 561401) RRID: AB_10645789
Thy1.1 (BD Biosciences; cat#, 557266) RRID: AB_396611
Thy1.1 (BD Biosciences; cat#, 741110) RRID: AB_2870702
CD45.1 (BD Biosciences; cat#, 561873) RRID: AB_1645214
CD62L (Tonbo; cat#, 35-0621) RRID: AB_2621697
CD62L (BD Biosciences; cat#, 563252) RRID: AB_2738098
CD44 (BD Biosciences; cat#, 560570) RRID: AB_1727486
CD44 (BD Biosciences; cat#, 560569) RRID: AB_1727484
TIGIT (eBioscience; cat#, 12-9501-80) RRID: AB_11039537
PD-1 (eBioscience; cat#, 12-9985-82) RRID: AB_466295
TIM3 (eBioscience; cat#, 12-5871-81) RRID: AB_465976
LAG-3 (eBioscience; cat#, 12-2231-82) RRID: AB_494214
KLRG1 (eBioscience; cat#, 12-5893-80) RRID: AB_10597431
CD25 (Invitrogen; cat#, MA5-17816) RRID: AB_2539200
TNF-alpha (BD Biosciences; cat#, 561041) RRID: AB_396761
IFNgamma (BD Biosciences; cat#, 563376) RRID: AB_2738165
IL-2 (BD Biosciences; cat#, 560544) RRID: AB_1645256
IL-18Rα (eBioscience; cat#, 12-5183-82) RRID: AB_2572617
IL-18Rα (eBioscience; cat#, 17-5183-82) RRID: AB_2744708
NKG2D (BD Biosciences; cat#, 562614) RRID: AB_2737677
CD49d (BD Biosciences; cat#, 564397) RRID: AB_2738789
T-bet (BD Biosciences; cat#, 564142) RRID: AB_2738616

NK1.1 (BioLegend; cat#, 108745) RRID: AB_2563286
CXCR3 (BioLegend; cat#, 126528) RRID: AB_2650922
4-1BB (BD Biosciences; cat#, 558976) RRID: AB_397172
CD69 (BD Biosciences; cat#, 553237) RRID: AB_394726
anti-mouse CD4 monoclonal antibody (BioXcell; cat#, BE0003) RRID: AB_1107642
anti-mouse MHC Class I (H-2) antibody (BioXcell; cat#, BE0077) RRID: AB_1125537
anti-mouse IL-18 monoclonal antibody (BioXCell; cat#, BE0237) RRID: AB_2687719

Eukaryotic cell lines

Policy information about cell lines

Cell line source(s)	B16-F10 was purchased from ATCC
Authentication	B16-F10 has been STR-authenticated by the Genome Analysis Team at the National Cancer Center.
Mycoplasma contamination	B16-F10 tested negative for mycoplasma contamination.
Commonly misidentified lines (See ICLAC register)	No misidentified cell lines were used in the study.

Animals and other organisms

Policy information about studies involving animals; ARRIVE guidelines recommended for reporting animal research

Laboratory animals	The study involved C57BL/6, CD45.1 congenic, Rag1 knockout, Il18r1 knockout, Foxp3-DTR and Thy1.1 Pmel-1 transgenic mice. Female mice were used at 6-8 weeks of age. All animals were maintained under specific pathogen-free conditions in the animal facilities of the National Cancer Center. Groups of up to five mice were housed in individually ventilated cages on a 12 h light/dark cycle, at an ambient temperature of 20 ± 2 °C, with humidity controlled at 55 ± 5 %.
Wild animals	No wild animals were used in the study.
Field-collected samples	No field-collected samples were used in the study.
Ethics oversight	Experimental procedures were approved by the Institutional Animal Care and Use Committee (IACUC) of the National Cancer Center Institute (NCCI). The NCCI animal facility is fully accredited by the Association for Assessment and Accreditation of Laboratory Animal Care International (AAALAC International). Animal experiments were conducted following the Guidelines on the Care and Use of Laboratory Animals from the Institute of Laboratory Animal Resources (ILAR).

Note that full information on the approval of the study protocol must also be provided in the manuscript.

Flow Cytometry

Plots

Confirm that:

- [x] The axis labels state the marker and fluorochrome used (e.g. CD4-FITC).
- [x] The axis scales are clearly visible. Include numbers along axes only for bottom left plot of group (a 'group' is an analysis of identical markers).
- [x] All plots are contour plots with outliers or pseudocolor plots.
- [x] A numerical value for number of cells or percentage (with statistics) is provided.

Methodology

Sample preparation	To prepare lymphocytes, lymph nodes and spleen were gently disrupted and filtered through a 40 μm nylon cell strainer (Falcon, NY, USA) to make single-cell suspensions. Cells were treated with RBC lysis buffer (eBioscience, CA, USA) before use. Tumor tissue-derived cells were prepared using a tumor dissociation kit (Miltenyi Biotec Inc., CA, USA) according to the manufacturer's instructions. Briefly, tumor tissues harvested from mice were cut into small pieces and transferred to gentle-MACS C tubes containing the enzyme mix. The mixture was processed using a gentleMACS dissociator (Miltenyi Biotec Inc.) and filtered through a 40 μm nylon cell strainer to obtain single-cell suspensions.
Instrument	Acquisition was performed using FACSCalibur and FACSVerse (BD Biosciences, NJ, USA). Fluorescence-activated cell sorting was performed using FACSAria and FACSMelody (BD Biosciences).
Software	Data were transferred and analysed using the FlowJo v10 (Tree Star Inc., OR, USA).
Cell population abundance	Cell population abundance within post-sort fractions is presented in Supplementary Fig. 3. The purity was determined by calculating the ratio of the desired cell fraction among the parent population.
Gating strategy	Live lymphocytes were gated by their FSC/SSC properties. Subsequent sub-population gating was performed by gating clearly distinguishable cell clusters. In the case of marker expression where the boundary was ambiguous, this was set to the

borderline that separates >99% of negative control (unstained). Detailed gating and sorting strategies are provided in Supplementary Fig. 3.

☒ Tick this box to confirm that a figure exemplifying the gating strategy is provided in the Supplementary Information.
